# Supplementary material for: Does the impact of open innovation depend on contextual factors? A case of the Korean biopharmaceutical industry
Source: PLoS One. 2024 Nov 19;19(11):e0310311. doi: 10.1371/journal.pone.0310311 (PMC11575768; doi:10.1371/journal.pone.0310311)
Supplement: Attachment — Submitted filename: [PlosOne]_Response to Reviewer #2_MAY24.docx [file pone.0310311.s002.docx]

**Response to Reviewer #2 Comments**

Thank you very much for all your comments. We are very glad that you give us the opportunity to revise our manuscript. We have worked hard to address your excellent and detailed comments. Based on them, we have substantially improved this manuscript. We hope you like the changes we made.

**Point 1:** The definition of Open Innovation, is presented only in page 4. Throughout the introduction no mention is reported on what the author refer to when talking about OI (for example in page 2 there is a reference on the fact that “OI is a broad concept defined in various ways” but no explication is reported)

**Response 1:** Thank you for your comment. As you pointed out, the exact definition of the concept of open innovation was missing although this paper deals with open innovation. A detailed explanation of OI concept has been added to the manuscript.

**Modification #1 (line 73 – line 93 in revised manuscript)**

Open innovation means intentionally allowing the inflow and outflow of knowledge into a firm to utilize external knowledge in value proposition design through a decentralized rather than centralized innovation process [7]. By including the financial and non-monetary benefits that can accrue to a variety of stakeholders, researchers are increasingly recognizing open innovation as a value co-creation process whose benefits extend beyond the enterprise [8]. Open innovation supports the establishment of a distributed innovation system in which companies open their internal innovation processes to external knowledge and technology [7, 8]. Unlike closed innovation systems, it also supports extending a firm's knowledge search strategy beyond its boundaries [9]. In this way, companies engage with customers [10], suppliers [11]and non-governmental organizations [11], involving various stakeholders, such as competitors, in a value creation strategy [8]. To integrate these stakeholders, companies can build a variety of engagement strategies across a variety of co-creation events and processes, such as crowdsourcing [12].

Open innovation consists of three forms: inbound, outbound, and combined [13]. The inbound open innovation process invites various external stakeholders to share information during the ideation and implementation stages. Relevant data are used to implement innovation through R&D processes [14]. In contrast, when companies engage with external stakeholders to more quickly send ideas to the market and commercially exploit available technological opportunities, the innovation process is called outbound open innovation [13]. Combined open innovation is a combination of inbound and outbound modalities. Dahlander and Gann [4] further divided open innovation types, classifying sourcing and acquisition as inbound and selling and disclosure as outbound open innovation. Abbate et al[15] stated that scholars often use the terms co-creation and open innovation as synonyms to refer to any type of creation achieved with all types of stakeholders. For example, value co-creation through strategic alliances or collaborations between companies is sometimes considered open innovation[8].

**Point 2:** Page 2, last paragraph: “Few studies have examined the relationship between an integrated perspective and the moderating factors in the impact of OI on technological innovation performance, and the results are inconsistent.” No work is mentioned to support such statement.

**Response 2:** Thanks for your sharp observation. In the process of organizing extant literature, reference citations were omitted. The phrase has been supplemented.

**Modification #1 (line 100 – line 104 in revised manuscript)**

In this way, open innovation serves as a tool to minimize business risks [16] and improve corporate performance, innovation performance quality [8], product scope and market share [11]. This is useful in companies that report the same variety of positive results. On the other hand, open innovation can also expose companies to significant risks during and after the innovation process [17]. Open innovation itself can lead to more unfavorable outcomes, such as knowledge outflow, and loss, compared to existing closed innovation [18].

**Point 3:** It is not clear in the text what is the difference between collaborations (one of the main covariates) and strategic alliances (one of the ‘mediating variables’).

“Strategic alliances are voluntary agreements between companies to develop and commercialize new products, technologies, or services” while - on the other hand - “This requires cooperation with external partners [26], meaning that the role of OI is increasing for companies. Though OI activities, that is, collaboration, companies can provide access to scarce knowledge and technologies, reduce development costs, provide risk-sharing possibilities, and improve product-development processes [27].”

There is need to provide more detail of the differences between the two and why you think you are capturing different things.

**Response 3:** Thanks for your insightful comment. According to your comment, two variables ‘*COLLABO*’ and ‘*ALLIANCE*’ are conceptually similar. It is necessary to clearly present the differences between them considering the purpose of research question. In the revised manuscript, the operational definition of each variable was revised to be more specific. Additionally, the reason why two conceptually similar variables were used was also added.

**Modification #1 (line 496 – line 502 in revised manuscript)**

Collaboration was based on domestic and foreign joint research in the relevant year, and strategic partnership was defined and used as the cumulative number of collaboration research. Firms secure and accumulate knowledge about alliance management by effectively utilizing and accumulating alliance experience [63]. Through alliance experience, they learn how to manage and consequently develop alliance management capability [64]. In other words, the more strategic experience, the more trackable the management capabilities are, so the cumulative number of strategic alliances was used as a proxy for alliance management capabilities.

**Modification #2 (line 228 – line 238 in revised manuscript)**

Bianchi et al. [29] stated that both technology and products are becoming more complex, and it is increasingly difficult for firms to independently participate in product and technology development, so the role of open innovation is increasing. Because the capabilities and knowledge required for such development are dispersed across firms and institutions (universities), collaboration is done with external partners [26]. The most important issue in collaboration between firms is the uncertainty of partner collaboration. Strategic alliance is the accumulated experience of such collaboration. Among various open innovation methods such as strategic alliance, joint venture, and M&A for R&D activities, strategic alliance is generally different from other methods. Considering the relatively low risks and costs compared to other methods, it is preferred by firms, and is the broadest form of collaboration that encompasses all forms of collaboration. Since strategic alliances are established for the strategic purposes of both firms promoting collaboration, they often involve one or more collaborations simultaneously in resource exchange, joint marketing, joint research and development, etc[39].

**Point 4:** Moreover, how the collaboration influences the cumulated stock of collaboration that should be linked to the strategic alliance management?

**Response 4:** As an extension of above response, this comment has also been modified.

**Modification #1 (line 369 – line 379 in revised manuscript)**

Alliance management capability can be defined as how much alliance experience has been secured and accumulated. Alliance management capability is the ability of a firm to capture knowledge about alliance management, share and store this knowledge, and apply this knowledge to current and future alliances [62]. Firms secure and accumulate knowledge about alliance management by effectively utilizing alliance experience and converting it into knowledge [63]. Firms learn to manage through alliance experience and consequently develop Alliance management capability [64]. In other words, Alliance management capability is based on alliance management knowledge gained through experience with various partners, which is useful for future alliances regardless of partner type. Therefore, alliance management capabilities allow partners to adapt the types of information and knowledge, shared understanding, and common goals shared within the alliance to changes in the environment, thereby improving performance [62]. The positive association between recent alliance experience and performance reflects the importance of dynamic capabilities

**Point 5:** The schematic diagram of research hypothesis need additional comment as it appears to be disjointed from the work.

**Response 5:** As you pointed out, the current figure (Fig 1) does not properly represent the research model. The figure has been modified into a more understandable form, and a detailed explanation has been provided in the manuscript.

**Modification #1 (line 444 – line 445 in revised manuscript)**

**Point 6:** Some details on how data has been collected are missing. For instance:

**Response 6:** Modifications to each comment are provided below.

- What does it mean that *“Data were constructed by referring to other accessible data as much as possible?”*

**Modification #1 (line 460 – line 464 in revised manuscript)**

In Korea, there is no site that provides all information about biopharmaceutical firm information. Corporate information (sales, firm size, etc.) was collected from KoDATA (KOREA RATING & DATA), information related to open innovation was collected from the website, Naver, and business reports, patent information was collected from the Korea Patent Information Service, and the number and amount of government R&D cases were collected from National Science & Technology Information Service

- What is the reason for excluding the biofood, biomedical device, biochemical/energy, and bioenvironmental industries?

**Modification #1 (line 468 – line 469 in revised manuscript)**

The goal of this study was limited to the biopharmaceutical industry, and heterogeneous characteristics were excluded in order to report only on biopharmaceutical companies.

- How passing from an initial number of 2,798 companies to 527 might influence your results.

**Modification #1 (line 806– line 807 in revised manuscript)**

In addition, out of the 2,798 firms surveyed, only 527 pharmaceutical and bio companies and companies with complete information were used, which was a limitation. Future research is needed to supplement the data.

- Moreover, among these firms, how many are included in the regressions that you show in the paper? As you should know, no information on the number of observations included in the regressions is provided to the reader.

**Modification #1 (line 703 in revised manuscript)**

Table 3. Results of 2SLS regression (N=527)

- The proxies for the variables included in the empirical work are not commented. They are reported only in the table 1.

**Modification #1 (line 540 – line 545 in revised manuscript)**

To verify the moderating effect of absorptive capacity of biopharmaceutical firms, R&D intensity was used as a proxy variable for absorptive capacity. It was calculated by dividing research and development costs by sales (Lin, Lee, & Hung, 2006). As a proxy for government R&D funds, the number of government R&D projects was used. There are many companies that carry out more than one government-supported R&D project per year, and the amount of funding may vary depending on the project in the data. For alliance management capabilities, the cumulative number of collaborations was used as an experience indicator of how many alliances there were.

**Point 7:** The results section is not sound.

**Response 7:** Modifications to each comment are provided below.

- A table with descriptive statistics of all the variables is missing (also the dependent ones and information on minimum and maximum values)

**Modification #1 (line 701 - 702 in revised manuscript)**

**Table 2. Descriptive statistics and correlations between variables**

|  | Mean | Std. Dev. | Min | Max | 1 | 2 | 3 | 4 | 5 | 6 | 7 | 8 | 9 | 10 | 11 | 12 |
| --- | --- | --- | --- | --- | --- | --- | --- | --- | --- | --- | --- | --- | --- | --- | --- | --- |
| *PATENT* | 2.035 | 4.237 | 0 | 48 | 1 |  |  |  |  |  |  |  |  |  |  |  |
| *TECH-TRANS* | 0.087 | 0.473 | 0 | 9 | 0.163 | 1 |  |  |  |  |  |  |  |  |  |  |
| *PURCHASE* | 0.103 | 0.465 | 0 | 7 | 0.147 | 0.228 | 1 |  |  |  |  |  |  |  |  |  |
| *COLLABO* | 0.322 | 0.923 | 0 | 10 | 0.236 | 0.245 | 0.239* | 1 |  |  |  |  |  |  |  |  |
| *ABSORP_CAP* | 6.131 | 18.207 | 0 | 187 | 0.456 | 0.337 | 0.236* | 0.280* | 1 |  |  |  |  |  |  |  |
| *GOV_SUP* | 0.984 | 1.621 | 0 | 12 | 0.330 | 0159 | 0.110* | 0.265* | 0.203* | 1 |  |  |  |  |  |  |
| *ALLIANCE* | 0.262 | 1.164 | 0 | 15 | 0.055 | 0.185 | 0.225* | 0.227* | 0.223* | 0.103* | 1 |  |  |  |  |  |
| *SIZE* | 62.651 | 81.073 | 0 | 1165 | 0.119 | 0.114 | 0.097* | 0.170* | 0.201* | 0.157* | 0.154* | 1 |  |  |  |  |
| *AGE* | 27.511 | 19.453 | 7 | 131 | 0.179 | 0.212 | 0.225* | 0.245* | 0.443* | 0.191* | 0.311* | 0.188* | 1 |  |  |  |
| *VENTURE* | 0.500 | 0.500 | 0 | 1 | 0.127 | 0.005 | -0.022 | 0.041* | -0.175* | 0.178* | -0.078* | -0.142 | -0.269* | 1 |  |  |
| *RND_CTR* | 0.813 | 0.405 | 0 | 5 | 0.245 | 0.117 | 0.130* | 0.188* | 0.117* | 0.255* | 0.113* | 0.012* | 0.179* | 0.555* | 1 |  |
| *DIVERSIFIC* | 2.059 | 1.232 | 1 | 4 | 0.222 | 0.192 | 0.224* | 0.241* | 0.369* | 0.214* | 0.292* | 0.344* | 0.534* | -0.128* | 0.280* | 1 |

- *“Technology purchasing did not have a positive effect on technological innovation performance, but technological cooperation exhibited a positive relationship (p<0.01), which indicates that the company has network partners”* this period does not make sense.

**Modification #1 (line 586 – line 592 in revised manuscript)**

Firm increase their external technical knowledge and strengthen their technical capabilities through technology purchases. The extent to which the acquisition of external technological knowledge affects a firm's performance depends on its internal R&D efforts, and Cohen and Levinthal (1990) stated that a firm 's internal development expertise improves the firm's absorptive capacity. In other words, companies with a high level of internal R&D can better integrate technologies acquired externally and achieve higher performance. Technical cooperation involves conducting joint research, etc. with partners, which means that a firm has a network partner.

- *“R&D intensity and government R&D support are positively related to technological innovation performance (all p<0.01), indicating that the biopharmaceutical industry is technology intensive”* does not make sense either.

**Modification #1 (line 595 – line 604 in revised manuscript)**

Government R&D subsidies have a positive effect on new product development and companies' patent performance (technological innovation performance). R&D intensity is defined as the internal organization's willingness to conduct research and development, that is, the ratio of total internal R&D expenditures to sales. In other words, through internal R&D investment, conflicts between technologies acquired from external organizations and the internal organization's technologies are minimized and complementary advantages are maximized to influence technological innovation performance. Technology-intensive industries should have higher R&D intensity than other industries. Companies must have high R&D of their own, and the greater the external financing, the more positive the impact. Technological innovation performance is important in technology-intensive industries. Government R&D support for the biopharmaceutical industry plays an important role in a firm 's technology investment and performance.

- p.19, incomplete sentence: *“This indicates that the performance of strategic alliances can be influenced by contingency factors depending on the characteristics of the partners, which may also differ because the purpose and function of each alliance relationship is [38]”*

**Modification #1 (line 616 – line 634 in revised manuscript)**

This is contrary to the common belief that the more experience in collaboration, the more active the collaboration with various entities. This means that even if there is a lot of experience in cooperation, it does not necessarily lead to technological innovation results. The biopharmaceutical industry incurs enormous development costs because the product development period from R&D to commercialization is relatively long compared to other industries [37]. Due to these industrial characteristics, strategic alliances between companies are increasing with various organizations such as universities, hospitals, government-funded research institutes, and biotechnology companies as well as pharmaceutical companies [14, 17]. The performance of these strategic alliances may differ because each type of alliance partner has different alliance motivations, resources and capabilities, organizational structure and culture, and degree of competition with partners (shin et al, 2016). Pharmaceutical and biotechnology firms and biotechnology firms acquire cutting-edge scientific knowledge through cooperation with research institutes and universities, and research institutes create scientific knowledge that can be used in industry (Siegel et al., 2003; Hung & Chiang, 2010 ). In other words, technological innovation performance can be improved by acquiring and learning professional knowledge through interaction with excellent researchers at research institutions and universities. On the other hand, partnerships between biotechnology firms and pharmaceutical firms can maintain R&D investments from pharmaceutical frims and utilize the pharmaceutical firm's resources and capabilities, such as facilities, personnel, and technology. These advantages improve technological innovation performance by promoting technology commercialization of biotechnology firms [10]. In addition, alliances between biotechnology firms are formed to create new technological innovation results by integrating the different technologies each possesses [16]. For this reason, high-tech firms are forming various alliances to secure external knowledge and accelerate technological innovation [52], but because the purpose and function of each alliance is different, contingency factors may be different [41] results are consistent with the results.

- The empirical exercise is not complete. No information on the number of observations or the goodness of fit is provided. No support to the need to adopt a negative binomial (e.g. showing a graph of the distribution of the patents) is provided. No additional statistics on the test of endogeneity of the instrumented variable is provided. It is not clear why inward OI variables – adopted in the first stage – are not adopted also in the second stage as they might provide insights on the technology transfer. Also an test on overidentification of the instruments would have been useful in order to appreciate the appropriateness of the empirical analysis.

**Modification #1 (line 554 – line 560 in revised manuscript)**

The number of registered patents, which is a dependent variable, is also a count variable, a non-negative integer that has multiple zeros. To utilize count data, the Poisson distribution or the negative binomial distribution must be considered in the analysis [73]. When using the Poisson model, the average and the variation of dependent variables should correspond to each other. According to previous studies, many count data display over-dispersion, meaning that the dispersion exceeds the average [74]. Over-dispersion is considered to be caused by unobserved heterogeneity, and as a frequent solution to this problem, it is assumed that the parameter of the Poisson distribution moves according to a specific probability distribution [75]. In this study, the average VIF was 3.64, as a result of the multicollinearity test. The detailed VIF results are summarized in the table below, and only the average VIF was briefly written and not reflected in the paper. (line 560)

| variables | Model 1 | | Model 2 | | Model 3 | | Model 4 | |
| --- | --- | --- | --- | --- | --- | --- | --- | --- |
|  | *VIF* | *1/VIF* | *VIF* | *1/VIF* | *VIF* | *1/VIF* | *VIF* | *1/VIF* |
| *SIZE* | 1.19 | 0.84 | 1.18 | 0.84 | 1.15 | 0.87 | 1.22 | 0.82 |
| *AGE* | 2.18 | 0.46 | 2.05 | 0.49 | 2.08 | 0.48 | 2.27 | 0.44 |
| *VENTURE* | 1.71 | 0.58 | 1.80 | 0.55 | 1.76 | 0.57 | 1.77 | 0.56 |
| *RND_CTR* | 1.48 | 0.68 | 1.51 | 0.66 | 1.51 | 0.66 | 1.48 | 0.68 |
| *DIVERSIFIC* | 1.96 | 0.51 | 1.86 | 054 | 1.87 | 0.53 | 1.97 | 0.51 |
| *TRANS* | 1.16 | 0.86 | 1.10 | 0.91 | 1.13 | 0.88 | 1.22 | 0.82 |
| *PURCHASE* |  |  |  |  |  |  | 14.05 | 0.07 |
| *COLLABO* |  |  |  |  |  |  | 2.62 | 0.38 |
| *ABSORP_CAP* | 2.15 | 0.47 |  |  |  |  | 2.21 | 0.45 |
| *GOV_SUP* |  |  | 1.33 | 0.75 |  |  | 1.35 | 0.74 |
| *ALLIANCE* |  |  |  |  | 2.38 | 0.42 | 2.52 | 0.40 |
| *PURCHASE × ABSORP_CAP* | 10.90 | 0.09 |  |  |  |  | 16.81 | 0.06 |
| *COLLABO × ABRORP_CAP* | 2.27 | 0.44 |  |  |  |  | 2.62 | 0.38 |
| *PURCHASE × GOV_SUP* |  |  | 1.88 | 0.53 |  |  | 3.40 | 0.29 |
| *COLLABO × GOV_SUP* |  |  | 2.04 | 0.49 |  |  | 1.95 | 0.51 |
| *PURCHASE × ALLIANCE* |  |  |  |  |  |  | 2.12 | 0.47 |
| *COLLABO × ALLIANCE* |  |  |  |  |  |  | 2.63 | 0.38 |
| *Mean VIF* | 3.40 | | 1.69 | | 1.63 | | 3.64 | |

- It is not clear why the purchase*alliance and collabo*alliance are included only in the model 4 and not – as one would expect coherently with models 1 and 2 – also in model 3. In general, to understand the existence of a ‘moderating’ effect of absorp_cap, gov_sup and alliance one would require to see also the effect of the estimates with only purchase and collabo variables.

**Modification #1 (line 703 in revised manuscript)**

Table 3. Results of 2SLS regression (N=527)

|  | Model 1 | | Model 2 | | Model 3 | | Model 4 | |
| --- | --- | --- | --- | --- | --- | --- | --- | --- |
|  | *PATENT* | *TRANSFER* | *PATENT* | *TRANSFER* | *PATENT* | *TRANSFER* | *PATENT* | *TRANSFER* |
| *PATENT* |  | 0.055^***^ (0.005) |  | 0.074^***^ (0.009) |  | 0.087^***^ (0.012) |  | 0.048^***^ (0.005) |
| *PURCHASE* | 0.017 (0.086) |  | 0.127^*^ (0.075) |  | 0.100^*^ (0.061) |  | 0.051 (0.100) |  |
| *COLLABO* | 0.247^***^ (0.042) |  | 0.253^***^ (0.044) |  | 0.219^***^ (0.034) |  | 0.268^***^ (0.057) |  |
| *ABSORP_CAP* | 0.021^***^ (0.002) |  |  |  |  |  | 0.018^***^ (0.002) |  |
| *GOV_SUP* |  |  | 0.184^***^ (0.015) |  |  |  | 0.167^***^ (0.017) |  |
| *ALLIANCE* |  |  |  |  | 0.017 (0.048) |  | -0.064^**^ (0.049) |  |
| *PURCHASE × ABSORP_CAP* | 0.001 (0.001) |  |  |  |  |  | 0.002^**^ (0.001) |  |
| *COLLABO × ABRORP_CAP* | -0.005^***^ (0.001) |  |  |  |  |  | -0.004^***^ (0.001) |  |
| *PURCHASE × GOV_SUP* |  |  | -0.029^*^ (0.022) |  |  |  | -0.034^**^ (0.025) |  |
| *COLLABO × GOV_SUP* |  |  | -0.030^***^ (0.010) |  |  |  | -0.030^*^ (0.012) |  |
| *PURCHASE × ALLIANCE* |  |  |  |  | -0.013(0.034) |  | -0.018 (0.034) |  |
| *COLLABO × ALLIANCE* |  |  |  |  | -0.002(0.034) |  | 0.029^**^ (0.041) |  |
| *SIZE* | 0.001 (0.001) | 0.001 (0.001) | 0.001^*^ (0.001) | 0.001 (0.001) | 0.001^***^ (0.001) | 0.001 (0.001) | 0.001^**^ (0.001) | 0.001 (0.001) |
| *AGE* | 0.001 (0.002) | 0.003^***^ (0.001) | 0.002^**^ (0.002) | 0.002^**^ (0.001) | 0.005^*^ (0.002) | 0.002^*^ (0.001) | -0.001^**^ (0.002) | 0.003^***^ (0.00) |
| *VENTURE* | 0.616^***^ (0.096) | 0.015 (0.030) | 0.472^***^ (0.084) | -0.001 (0.028) | 0.609^***^ (0.086) | -0.009 (0.029) | 0.463^***^ (0.096) | 0.018 (0.030) |
| *RND_CTR* | 0.385^***^ (0.098) | -0.019 (0.034) | 0.479^***^ (0.076) | -0.037 (0.032) | 0.464^***^ (0.081) | -0.048 (0.034) | 0.381^***^ (0.093) | -0.015 (0.034) |
| *DIVERSIFIC* | 0.111^***^ (0.039) | 0.003 (0.012) | 0.121^***^ (0.033) | 0.004 (0.012) | 0.114^***^ (0.034) | 0.001 (0.012) | 0.130^***^ (0.038) | 0.005 (0.013) |

Notes: ^***^, ^**^, ^*^ represents p<0.01, p<0.05, p<0.1, respectively. Standard errors are between brackets next to the coefficient.

- Some concerns on the interpretation of the results arise as well. For instance, in page 19 the author writes The results support the findings of Zhang and Guan [63] and Shin et al. [37], providing that government R&D support does not have a positive effect or may have a negative effect on a company’s technological innovation performance. However, the impact of gov.support on technological innovation is positive (model 2 and 4). What is negative is the interaction with the variables purchase and collabo. This indicates that, although positively associated with the dependent variable, government support is concave with respect to purchase and collabo and vice versa purchase and collabo are concave with respect to government support.

**Modification #1 (line 647 – line 651 in revised manuscript)**

Government R&D subsidy had a positive effect on companies' technological innovation performance. However, the government's R&D support, which is a moderating factor, had a negative moderating effect on technology purchase and technological cooperation (p<0.05, p<0.1, separate). Thus, additionality through public support, as a proxy for government R&D support, activates the limits of inbound OI on technological innovation performance (Hypothesis 2 is not supported).
