# Supplementary material for: Does the impact of open innovation depend on contextual factors? A case of the Korean biopharmaceutical industry
Source: PLoS One. 2024 Nov 19;19(11):e0310311. doi: 10.1371/journal.pone.0310311 (PMC11575768; doi:10.1371/journal.pone.0310311)
Supplement: Attachment — Submitted filename: [PlosOne]_Response to Reviewer #1_JUL24.docx [file pone.0310311.s003.docx]

**Response to Reviewer #1 Comments**

**Point 1:** I have carefully reviewed the revised manuscript submitted by the authors. They have addressed all my previous comments and concerns comprehensively and have made substantial improvements to the clarity, methodology, and overall presentation of the paper. The authors’ responses were thorough and satisfactory, and the revisions have strengthened the manuscript significantly. Therefore, I recommend accepting the manuscript for publication.

**Response 1:** Thank you for your recommendation for publication. We believe that your valuable comments have helped us to improve the manuscript.
