# Supplementary material for: Does the impact of open innovation depend on contextual factors? A case of the Korean biopharmaceutical industry
Source: PLoS One. 2024 Nov 19;19(11):e0310311. doi: 10.1371/journal.pone.0310311 (PMC11575768; doi:10.1371/journal.pone.0310311)
Supplement: Attachment — Submitted filename: [PlosOne]_Response to Reviewer #2(3rd)_AUG24.docx [file pone.0310311.s004.docx]

**Response to Reviewer #2 Comments (3^rd^ Round)**

Thank you very much for all your comments. We are very glad that you give us the opportunity to revise our manuscript. We have worked hard to address your excellent and detailed comments, to supplement the shortcomings of our article. Based on them, we have substantially improved this manuscript. We hope you like the changes we made.

**Point 1:** no reference or comment in the text on the diagram reported in figure 1 is reported despite the author's comment stating otherwise.

**Response 1:** Thank you for your comment. As pointed out, this paper reflects the references or explanations in the text of the diagram reported in Figure 1. In this study, the research hypothesis is shown in Figure 1. In the figure, this study investigates the relationship between inbound open innovation (technology purchase and technology collaboration) and innovation performance (patents) of biopharmaceutical firms, and diagrams what moderating factors(Government R&D support, Absorptive capacity, Alliacne management capability) are related to innovation performance (patents) and outbound open innovation (technology transfer).

**Modification #1 (line 235, 277-278, 324, 373, 428, 462, line 467- 544 in revised manuscript)**

**Figure 1. Schematic diagram of research hypothesis**

The above figure structures the study's hypotheses in Figure 1. This study investigates the relationship between inbound open innovation (technology purchasing and technology collaboration) and innovation performance (patents) of biopharmaceutical firms, and what moderating factors are related between innovation performance (patents) and outbound open innovation (technology transfer). Technology purchasing is important for R&D-intensive (high-tech) firms because they have a high demand for innovation [36]. Firms can attract R&D investment through technology purchases and utilize other organizations’ resources and capabilities, including technology [38], It can develop internal innovation processes by integrating and utilizing external prepared technologies to address market gaps [42]. Technology purchasing can improve sales generation and financial performance by shortening the time for new product development and creating innovation outcomes [40], as it allows companies to develop complex products through the integration of proven technologies [41].

This requires cooperation with external partners [45], meaning that the role of OI is increasing for firms. Through OI activities, that is, collaboration, firms can provide access to scarce knowledge and technologies, reduce development costs, provide risk-sharing possibilities, and improve product-development processes [46]. Collaboration represents a distinct type of open innovation because it involves mutual innovation activities with common goals and the active participation of external stakeholders [71]. High-tech firms engage in extensive collaboration to secure external knowledge and accelerate technological innovation. Furthermore, as the capabilities and knowledge required for development are distributed across firms and institutions (universities), they collaborate with external partners. Collaboration increases the likelihood of goal achievement by securing additional resources and avoiding negative contingencies [48]. Such collaboration promotes innovation as a driving force for knowledge production and the creation of new innovations and expands a firm’s knowledge base that can be exploited for knowledge redistribution or transfer.

In particular, in the biopharmaceutical industry, open innovation is no longer an option but an essential strategic measure. Pisano [14] finds the reason in the characteristics of biopharmaceutical [14]. First, biopharmaceutical has high uncertainty, second, it has multidisciplinary characteristics, and third, it requires technological accumulation. Because of the high uncertainty of technology, biopharmaceutical firms proceed with one or two highly certain candidate substances in the pipeline, and also do not solve all processes from candidate substance discovery to clinical trial performance on their own, but rather choose to divide the work by stage. Furthermore, since biopharmaceutical firms often base their efforts on one or two accumulated core technologies, they must integrate complementary technologies from various fields through open innovation in order to develop them into commercial technologies [36]. In other words, because pharmaceutical and biotechnology requires accumulated capabilities through numerous failures, it is impossible to develop technologies from various fields together, which means that open innovation is inevitable. These industrial characteristics provide pharmaceutical and biotechnology firms with strategic advantages such as avoiding high costs for internal development, achieving rapid growth [42], and accessing cutting-edge technologies through technology purchases and collaborations with various research institutes such as universities, hospitals, and government-funded research institutes as well as pharmaceutical firms [19, 23]. Therefore, biopharmaceutical firms can select excellent technologies from biotechnology firms in advance, reduce the risk of failure during development, and thereby increase the R&D efficiency of biotechnology firms, thereby promoting technological innovation results.

In addition, through this opportunity to acquire and learn complementary resources and capabilities from external organizations, biopharmaceutical firms can receive positive impacts, such as R&D performance and patent performance [24]. In addition, excellent scientific knowledge or basic technology from research institutes and biotechnology firms is transferred to pharmaceutical firms, which helps in terms of commercialization, improved financial performance, and technological innovation performance. It is schematized. In addition, this study hypothesizes the moderating effects of government R&D support, absorptive capacity, and alliance management capacity on the relationship between inbound open innovation and innovation performance, and the relationship between innovation performance and outbound open innovation (technology transfer) as a moderating factor. set. Since innovation depends on a firm's ability to make external linkages and manage the innovation process [8], we propose that two specific types of organizational capabilities, namely alliance management capabilities and absorptive capabilities, will affect innovation performance. The government's R&D support serves as a source of funds for initial technology investment by firms with insufficient funds, promotes external cooperation or financing, and indirectly strengthens the company's R&D alliance by strengthening the company's absorptive capacity [77, 78]. On the other hand, companies that receive government R&D support have a crowding-out effect in which firms own R&D investment is replaced by government R&D support, showing a negative relationship [79]. Additionally, there is a possibility of moral hazard in using government R&D support for purposes other than research and development. There are also studies that show that there is no significant relationship between government R&D support and innovation performance. Therefore, efforts are needed to break away from the mixed results between government support and innovation performance and find better outcome variables that can verify the effectiveness of government R&D support. Additionally, strengthening a firm’s absorptive capacity increases the possibility of strategic alliances with various organizations [80]. Therefore, this study investigated how absorptive capacity, government R&D support, and alliance management capacity influence the moderating factors in the relationship between open innovation and performance, focusing on the Korean biopharmaceutical industry, a catch-up country.

**Reference**

8. Chesbrough, H., & Bogers, M. Explicating open innovation: Clarifying an emerging paradigm for understanding innovation. New Frontiers in Open Innovation. Oxford: Oxford University Press, Forthcoming, 2014; 3-28.

14. Lyu, J., Wang, S., Balius, T. E., Singh, I., Levit, A., Moroz, Y. S., ... & Irwin, J. J. Ultra-large library docking for discovering new chemotypes. Nature.2019;566:, 224-229.

19.	Cohen WM, Levinthal DA. Absorptive capacity: A new perspective on learning and innovation. Admin Sci Q. 1990;35: 128-152. doi: 10.2307/2393553

23.	Kang KN, Park H. Influence of government R&D support and inter-firm collaborations on innovation in Korean biotechnology SMEs. Technovation. 2012;32: 68-78. doi: 10.1016/j.technovation.2011.08.004.

24.	Baum JAC, Calabrese T, Silverman BS. Don't go it alone: Alliance network composition and startups' performance in Canadian biotechnology. Strateg Manag J. 2000;21: 267-294. doi: 10.1002/(SICI)1097-0266(200003) 21:3<267::AID-SMJ89>3.0.CO;2-8.

36.	Rothaermel FT, Deeds DL. Alliance type, alliance experience and alliance management capability in high-technology ventures. J Bus Venturing. 2006;21: 429-460. doi: [10.1016/j.jbusvent.2005.02.006](https://doi.org/10.1016/j.jbusvent.2005.02.006).

38.	Rothaermel FT. Complementary assets, strategic alliances, and the incumbent’s advantage: An empirical study of industry and firm effects in the biopharmaceutical industry. Res Policy. 2001;30: 1235-1251. doi: 10.1016/S0048-7333(00)00142-6.

40.	Wang Y, Vanhaverbeke W, Roijakkers N. Exploring the impact of open innovation on national systems of innovation — A theoretical analysis. Technol Forecasting Soc Change. 2012;79: 419-428. doi: 10.1016/j.techfore.2011.08.009.

41.	Van de Vrande V, De Jong JPJ, Vanhaverbeke W, De Rochemont M. Open innovation in SMEs: Trends, motives and management challenges. Technovation. 2009;29: 423-437. doi: 10.1016/j.technovation.2008.10.001.

42.	Chesbrough H, Vanhaverbeke W, West J, editors. Open innovation: Researching a new paradigm. Oxford University Press; 2006.

45. Frishammar J, Parida V, Westerberg M. Inbound open innovation activities in high‐tech SMEs: The impact on innovation performance. J Small Bus Manag. 2012;50: 283-309. doi: 10.1111/j.1540-627X.2012.00354.x.

46.	Henkel J. Selective revealing in open innovation processes: The case of embedded Linux. Res Policy. 2006;35: 953-969. doi: 10.1016/j.respol.2006.04.010

48.	Audretsch DB, Belitski M. The role of R&D and knowledge spillovers in innovation and productivity. Eur Econ Rev. 2020;123: 103391. doi: 10.1016/j.euroecorev.2020.103391.

71.	Enkel E, Gassmann O, Chesbrough H. Open R&D and open innovation: Exploring the phenomenon. R D Manag. 2009;39: 311-316. doi: 10.1111/j.1467-9310.2009.00570.x.

77. Sakakibara, M. Heterogeneity of firm capabilities and cooperative research and development: an empirical examination of motives. Strategic management journal. 1997;18:143-164. doi: 10.1002/(sici)1097-0266(199707)18:1+ <143::aid-smj927>3.0.co;2-y

78. Svensson, R. Innovation performance and government financing. Journal of small business & Entrepreneurship.2008; 21: 95-116. doi:10.1080/08276331.2008.10593415

79. Luukkonen, T.The difficulties in assessing the impact of EU framework programmes. Research Policy. 1998; 27: 599-610. doi:10.1016/s0048-7333(98)00058-4

80. Muscio, A. The impact of absorptive capacity on SMEs' collaboration. Economics of Innovation and New Technology. 2007;16: 653-668. doi:10.1080/10438590600983994

**Point 2:**

**Point 2a:** the empirical part is still poorly sound: the only innovation reported in this round is the performance of the VIF test for testing variables' collinearity which was not the point of the previous review. The methodology is a IV regression with count dependent variable. The reader needs to know that, for instance, a overdispersion test (to show the preferrability of negative binomial vs poisson) would be informative.

**Response 2a:** Thanks for your comment. In our study, we removed the performance results of the VIF test for testing the collinearity of variables. In addition we conducted an overdispersion test (to show the preference of negative binomial vs. Poisson) in the methodology. The results reflect the basis and conclusion that the negative binomial regression is an appropriate analysis model. We additionally reflected the content and results you mentioned in the manuscript. **(line 663 – line 693 in revised manuscript)**

In this study, negative binomial regression and Poisson binomial regression tests were performed, and it was determined that negative binomial regression analysis was an appropriate analysis model because the log likelihood value (-2063.3818), AIC value (4164.764), and BIC value (4261.851) were smaller than those of the Poisson analysis results. The detailed methods and results are as follows.

The Poisson regression rarely fits in practice since in most applications the conditional variance is greater than the conditional mean. If the mean structure is correct, but inefficient (Gourierourx et al., 1984). Further, the standard errors from the Poisson regression model will be biased downward, resulting in spuriously large z-values (Cameron and Trivedi 1986, p. 31).

In this study, Negative binomial regression and Poisson binomial regression tests were performed to obtain information on the goodness of fit of the two stages of the IV regression. The negative binomial regression results showed that the Log likelihood value was –2063.3818, the AIC (Akaike Information Criterion) was 4164.764, and the BIC (Bayes Information Criterion) was 4261.851. The Poisson binomial regression results showed that the Log likelihood value was –2084.8472, the AIC (Akaike Information Criterion) was 5783.694, and the BIC (Bayes Information Criterion) was 5870.562.

Also, the likelihood-ratio test is a test for the overdispersion parameter alpha (our results show that the Likelihood-ratio test of alpha=0: chibar2(01) = 1558.42 Prob>=chibar2 = 0.000). When the overdispersion parameter is 0, the negative binomial distribution is identical to the Poisson distribution. In this case, alpha is significantly different from 0, so we emphasize once again that the Poisson distribution is not appropriate. In other words, Testing for Overdispersion (a test to statistically justify why Negative binomial regression vs Poisson binomial regression model is used) rejected Ho within a statistically significant range, so NBR was used rather than the Poisson regression model.

AIC (Akaike Information Criterion) and BIC (Bayes Information Criterion) are standard measures for model selection, and a regression equation with a smaller value, whether it is the AIC value or the BIC value, is a more appropriate regression equation. In other words, a smaller AIC and BIC means that the model has the largest degree of coupling (likelihood) and the smallest number of variables. In this study, considering the analysis results, negative binomial regression was judged to be an appropriate analysis model.

**Point 2b:** No information on the goodness of fit of the two stages of the IV regression is provided. No test on the instruments adopted is available.

**Response 2b:** Thanks for your sharp observation. We additionally reflected the content and results you mentioned in the manuscript. **(line 694 – line 706 in revised manuscript)**

In addition, after performing 2SLS, an estimation method using instrumental variables (IV) was implemented, and the F test statistic was less than 10 (F test statistic = 3.72096), so it was judged that the correlation with the endogenous variable was weak. The detailed explanation and method are as follows.

In this study, an estimation method using instrumental variables (IV) was implemented after performing 2SLS. Instrumental variables must not necessarily be correlated with the error term of the regression model and must be correlated with the endogenous explanatory variables. In IV estimation, the number of instrumental variables must be greater than or equal to the number of endogenous explanatory variables, and when there are more instrumental variables than endogenous explanatory variables, it is called over-identification. In the case of over-identification, it is necessary to test the validity of the instrumental variables, that is, to conduct an over-identification test. Therefore, in this study, an over-identification test was additionally conducted after 2SLS. If the F test statistic is greater than 10, it can be judged that there is a correlation with the endogenous variables. In the results of our study, the F test statistic = 3.72096, indicating a weak correlation. The Sagan and Basmann p-values (p = 0.0916, p = 0.0938) of the over-identification test are greater than 0.05, so the instrumental variables are not correlated with the error term at the 5% significance level.

**Point 3:** the information added in response to previous point 2 needs a revision. In the paragraph reported below, the first 2/3 periods need references:

"Few studies have examined the relationship between an integrated perspective and the moderating factors in the impact of OI on technological innovation performance, and the results are inconsistent. Firm must invest resources to absorb knowledge outflow. Among invested resources, internal R&D investment has been considered the most important resource for creating new knowledge and absorptive capacity. Internal R&D investment refers to the extent to which a firm invests in internal activities (e.g., new product development) and research and development resources, and internal R&D investment is said to be important in developing technological knowledge that contributes to absorptive capacity [19]."

**Response 3:** Thanks for your sharp observation. There are few studies investigating the relationship between the integrated perspective and moderating factors in the impact of OI on technological innovation performance, and the results were inconsistent, so we revised the content to reflect this. There are few studies that have investigated the relationship between the integrated perspective and moderating factors in the impact of OI on technological innovation performance, and the results are inconsistent, so we revised and reflected the content. In addition, we summarized existing studies on the types of open innovation, open innovation incentives, open innovation within the value chain, and the relationship between these and technological innovation performance from a strategic perspective in the biopharmaceutical industry. We summarized and supplemented the various existing studies related to open innovation in biopharmaceutical firms. In addition, we have organized and reflected references from existing research that have been secured.

**Modification #1 (line 96 – line 152 in revised manuscript)**

Existing studies related to open innovation in the biopharmaceutical industry categorize open innovation types from a strategic perspective, open innovation incentives [20, 21], open innovation targets in the value chain, and The resulting relationship with technological innovation performance [22, 23, 24], the complementary resources or partnership experience of the two companies, differences in knowledge base, absorptive capacity, and government R&D support. The differences in performance and the choice of open innovation type were emphasized [19, 25, 26, 27, 28, 29].

In a study by Bianchi et al [20], depending on the stage of the development process, biopharmaceutical companies aim to acquire technology and knowledge through licensing agreements, alliances, etc. (inbound open innovation) or utilize it commercially (outbound open innovation). They said they are establishing increasingly intensive relationships with a variety of partners (e.g. large pharmaceutical companies, biotechnology companies, universities, etc.). A study by Allarakhia et al [21] found that open knowledge networks and other collaborative strategies give biopharmaceutical companies access to immaterial knowledge-based resources that are important for downstream drug development, and that these collaborative strategic alliances enable researchers to develop commercial products. When production is impossible and the costs associated with excessive upstream competition are too high, companies can jointly obtain incentives through collaborative knowledge production and open knowledge dissemination.

A study by Wang & Zajac [25] found that in the biopharmaceutical industry, the higher the similarity in resources and capabilities between two companies, the more likely it is to trigger companies to choose an acquisition as a governance form of resource combination rather than an alliance. A study by Shin et al [22] empirically analyzed the impact on technological innovation performance by type of alliance partner of biopharmaceutical companies and classified strategic alliances for R&D activities in the biopharmaceutical industry into three types to determine absorption capacity and potential competition. The moderating effect was identified. Vertical alliances have a positive effect on technological innovation performance, horizontal alliances have been shown to have an inverted U-shaped relationship with technological innovation performance due to the influence of competition, and the R&D intensity of biotechnology companies has a positive effect on technological innovation performance. -It was confirmed that there is a moderating effect that increases the impact of upstream alliances. A study by Baum et al [24] investigated the impact of changes in the alliance network composition of Canadian biotechnology startup companies on initial performance. They suggest that startups can improve their initial performance by building alliances, organizing them into efficient networks that provide access to diverse information and capabilities with minimal redundancy, conflict, and complexity costs, and by carefully forming alliances with potential competitors. . A study by Kang & Park [23] investigated the effect of cooperation between pharmaceutical and bio companies and the direct and indirect effects of government R&D support on innovation performance. According to the research results, upstream partnerships were significantly related to companies' innovation performance, and government R&D support directly and indirectly affected companies' innovation by promoting internal R&D and domestic upstream and downstream cooperation. The importance of government R&D support and networking and cooperation between universities, research institutes, and subparts was emphasized. A study by Carayannopoulos, & Auster [30] found that biopharmaceutical companies are more likely to source external knowledge through acquisition when the knowledge area is more complex and valuable when choosing acquisition or alliance when sourcing external knowledge, and that there is a higher possibility of sourcing external knowledge through alliance. The relationship between the two was also said to be strengthened.

Lin et al [26] investigated the impact on innovation performance from the perspective of inter-firm R&D alliance experience and absorptive capacity as an essential mechanism for creating new technological knowledge. Firms with high absorptive capacity and firms with more alliance experience show more innovative performance, and in particular, innovation performance peaks when the technological distance from the alliance partner is at a medium level when interacted with the proportion of R&D alliances in the firm's alliance portfolio. did. In addition, it was said that R&D alliances complement rather than replace internal R&D within a company. A study by Xia & Roper [27] investigated the impact of the relationship between absorptive capacity and external relationships, two key aspects of open innovation, on the growth of small biopharmaceutical companies in the United States and Europe. Research results show that absorptive capacity plays an important role in a company's growth, and that exploratory relationships are largely dependent on the continuity of R&D in terms of a company's absorptive capacity and interaction with the outside world. On the other hand, participation in exploitative relationships is related to the company's absorptive capacity. It was said that there were more conditions regarding competency.

According to George et al [28], pharmaceutical and bio companies' alliance portfolio characteristics and absorptive capacity together affect the company's innovative and financial performance. Lu et al [29] classified them into inbound OI and outbound OI, respectively. The impact on a company's innovation performance was studied. Research results show that inbound and outbound OI have a positive effect on a company's innovation performance, and absorptive capacity positively regulates inbound and outbound OI and a company's innovation performance. As such, a variety of existing studies have been conducted on open innovation in biopharmaceutical companies, but the studies that have been conducted so far are fragmented and do not present integrated results. In addition, existing studies were mainly conducted in advanced countries in the biopharmaceutical industry, necessitating caution in interpreting the implications of open innovation in catching-up countries. In this regard, this study investigated how absorptive capacity, government R&D support, and alliance management capacity affect the moderating factors in the relationship between open innovation and performance, focusing on the Korean pharmaceutical and bio industry, a catch-up country. We aim to derive managerial and policy implications for open innovation in the biopharmaceutical industry by applying it to Korean companies, which are catching up in the biopharmaceutical industry, and analyzing and comparing them.

**Reference**

20. Bianchi, M., Cavaliere, A., Chiaroni, D., Frattini, F., & Chiesa, V. Organisational modes for Open Innovation in the bio-pharmaceutical industry: An exploratory analysis. Technovation, 2011;31:22-33. doi:10.1016/ j.technovation.2010.03.002

21. Allarakhia, M., Marc Kilgour, D., & David Fuller, J. Modelling the incentive to participate in open source biopharmaceutical innovation. R&d Management, 2010;40: 50-66. doi: 10.1111/j.1467-9310.2009.00577.x

22. Shin, K., Kim, E., & Jeong, E. Structural Relationship and Influence between Open Innovation Capacities and Performances. Sustainability, 2018;10: 27-87. doi: 10.3390/su10082787

23.	Kang KN, Park H. Influence of government R&D support and inter-firm collaborations on innovation in Korean biotechnology SMEs. Technovation. 2012;32: 68-78. doi: [10.1016/j.technovation.2011.08.004](https://doi.org/10.1016/j.technovation.2011.08.004).

24.	Baum JAC, Calabrese T, Silverman BS. Don't go it alone: Alliance network composition and startups' performance in Canadian biotechnology. Strateg Manag J. 2000;21: 267-294. doi: 10.1002/(SICI)1097-0266(200003) 21:3<267::AID-SMJ89>3.0.CO;2-8.

25. Wang, L., & Zajac, E. J. Alliance or acquisition? A dyadic perspective on inter firm resource combinations. Strategic management journal, 2007;28: 1291-1317. doi: 10.1002/smj.638

26. Lin, C., Wu, Y. J., Chang, C., Wang, W., & Lee, C. Y. The alliance innovation performance of R&D alliances—the absorptive capacity perspective. Technovation, 2012;32: 282-292. doi: 10.1016/j.technovation.2012.01.004

27. Xia, T., & Roper, S. Unpacking open innovation: Absorptive capacity, exploratory and exploitative openness, and the growth of entrepreneurial biopharmaceutical firms. Journal of Small Business Management. 2016; 54: 931-952. doi: 10.1111/jsbm.12220

28. George, G., Zahra, S. A., Wheatley, K. K., & Khan, R. The effects of alliance portfolio characteristics and absorptive capacity on performance: A study of biotechnology firms. The Journal of High Technology Management Research,2001;12: 205-226. doi: 10.1016/s1047-8310(01)00037-2

29. Lu, C., Qi, Y., & Hao, S. Enhancing innovation performance of SMEs through open innovation and absorptive capacity: the moderating effect of business model. Technology Analysis & Strategic Management, 2023;1-17.
